# Prevalence of and Factors Associated with Club Drug Use among Secondary Vocational Students in China

**DOI:** 10.3390/ijerph181910408

**Published:** 2021-10-03

**Authors:** Jincong Yu, Qingfeng Wu, Yuqin Wu, Jiang Li, Qinxuan Wu, Huiping Cao, Zengzhen Wang

**Affiliations:** 1 Psychological Health Education and Counseling Center, Zhongnan University of Economics and Law, Wuhan 430073, China; jincongyu2016@zuel.edu.cn; 2 School of Public Health and Health Management, Gannan Medical University, Ganzhou 341000, China; wuqf2006@gmu.edu.cn; 3 School of Foreign Languages, Zhongnan University of Economics and Law, Wuhan 430073, China; wuyuqin1224@zuel.edu.cn; 4 Chongqing Health Statistics Information Center, Chongqing 401120, China; lijiangchres@163.com; 5 Guangdong Province Technician College of Light Industry, Guangzhou 510315, China; wuqinxuan0329@163.com; 6 Zhaoqing Secondary Vocational School of Science and Technology, Zhaoqing 526020, China; caohuiping166@163.com; 7 Department of Epidemiology and Biostatistics, School of Public Health, Tongji Medical College, Huazhong University of Science and Technology, Wuhan 430030, China

**Keywords:** club drug use, prevalence, associated factors, secondary vocational students, China

## Abstract

To understand the prevalence of and factors associated with club drug use among Chinese secondary vocational students, a nationally representative survey was conducted. The multistage cluster sampling strategy was employed to select participants. A total of 9469 students from eleven secondary vocational schools in five cities completed self-reported questionnaires, which included information on club drug use, sociodemographic variables, individual factors, as well as peer and family related factors. The data were separately analyzed with Poisson regression models for female and male students. The overall lifetime prevalence of club drug use was 2.7% (258/9469), and male students had higher prevalence than female students (3.5% vs. 1.9%, *p* < 0.001). Female and male students shared four risk factors (i.e., having ever smoked, perceiving social benefit expectancies, peer drug using and perceiving peer’s approval of drug use) and one protective factor (i.e., having medium or high levels of refusal skills) for club drug use. Moreover, family drug using and having a part-time job were two additionally independent risk factors for club drug use among male students. These findings indicate that the problem of club drug use among Chinese secondary vocational students is worthy of attention. The prevention of club drug use should address multiple risks and protective factors on individual, peer and family levels.

## 1. Introduction

Drug use is a serious public health problem worldwide. As shown in the World Drug Report 2020 [1], approximately 269 million people, which accounted for 5.4% of the global population aged 15–64 years, had used drugs (including opioids, cannabis, ecstasy, methamphetamine, etc.) in 2018. Drug use had globally resulted in 585,000 deaths and 42 million years of “healthy” life lost in 2017 [2]. Moreover, the global burden of disease attributable to drug use was 1.8%, and drug use was ranked as the sixth highest in terms of disease burden among young people aged 10–24 years in 2019 [3]. Furthermore, according to a report on the Chinese drug situation in 2019, cumulative total registered drug abusers reached 2.9 million, which accounted for 0.16% of the total Chinese population, 49% of whom were under 35 years old, as well as 0.3% under 18 years old [4].

Adolescence is an important period of physical, cognitive and emotional development, with robust behavioral, morphological, hormonal, and neurochemical changes [5]. It is also a vulnerable period of substance use, having an especially high risk for the initiation of substance use [6]. As shown in the European School Survey Project on Alcohol and Other Drugs (ESPAD), the lifetime prevalence of illicit drug use was 17.0% among European students aged 15–16 in 2019, with 16.0%, 2.3%, 1.7% and 0.7% for cannabis, ecstasy, methamphetamine and gamma-hydroxybutyrate (GHB), respectively [7]. According to the results of the Global School-Based Student Health Survey (GSHS) from different regions around the world, the prevalence of past-month cannabis use ranged from 3.1% to 15.5%, and the prevalence of lifetime amphetamine use ranged from 1.0% to 14.5% among young people aged 13 to 17 years [8,9,10,11,12]. Jia, Z., et al. reviewed 72 studies and reported that the pooled prevalence of illicit drug use was 2.1% among students in mainland China, with the prevalence ranging from 0.4% to 4.2% in different provinces [13].

Club drugs are a diverse group of recreational drugs that are used primarily by teenagers and young adults at raves, dance parties, nightclubs, and concerts [14]. Club drugs have been used more and more by younger Chinese people [15,16]. In China, five popular club drugs are Ketamine, methamphetamine (MA), Ecstasy (MDMA), ‘Magu’ pills (capsules which usually mix MA with caffeine) and GHB [17,18]. Club drugs can cause substantial physical and mental damage, such as vomiting, amnesia, delirium, aggression, anxiety, depression, suicidal ideation, psychotic episodes, and so on [19,20,21]. Their use is also closely correlated with high-risk sexual behavior and HIV transmission [17], as well as sexual assaults [22]. Moreover, their use may elevate the risk of violence, injuries and aberrant driving [23]. The most serious problem is that an overdose of drug use can also result in fatal cases [22,24].

To address these adverse consequences, it is imperative to take measures to prevent club drug use. Identifying the risks and protective factors associated with club drug use is crucial for the development of prevention programs [25,26]. Previous studies [6,16,27,28] have established the relationship between a series of correlates with drug use behavior. These correlations include sociodemographic factors (e.g., gender, age, social economics status), lack of knowledge about drugs, personality traits (e.g., impulsivity, sensation-seeking), peer drug use, family factors (e.g., family drug use, parental monitoring), school factors (e.g., neglecting the drug prevention education, academic pressure), social environmental factors (e.g., availability of drugs, subculture) and circadian rhythm.

The vocational education is an important part of upper secondary education in China. Chinese secondary vocational students receive a three-year vocational/technical curriculum after graduating from junior high schools [29]. The Ministry of Education of the People’s Republic of China announced that there were ten thousand secondary vocational schools with 15.8 million students in 2019, which accounted for 39.5% of senior high school students [30]. Though most students were generally 15–18 years old in secondary vocational schools, there were also a few older students for suspension and return [31]. Previous studies [31,32,33,34] have shown that secondary vocational students had a higher prevalence of illicit drugs use than other types of school students. Nevertheless, studies of club drug use among Chinese secondary vocational students are limited. From my perspective, only a limited number of studies have reported on the prevalence and associated factors of club drug use among Chinese secondary vocational students [34,35,36]. However, these samples were all regional. Hence, the results from this prior research does not completely represent the current status of secondary vocational student’s club drug use in China. The present study is positioned to fill this knowledge gap, with the goal of understanding the lifetime prevalence of club drug use among Chinese secondary vocational students and determining the risks and protective factors by a nationally representative sample.

## 2. Materials and Methods

### 2.1. Participants

**Figure 1:**
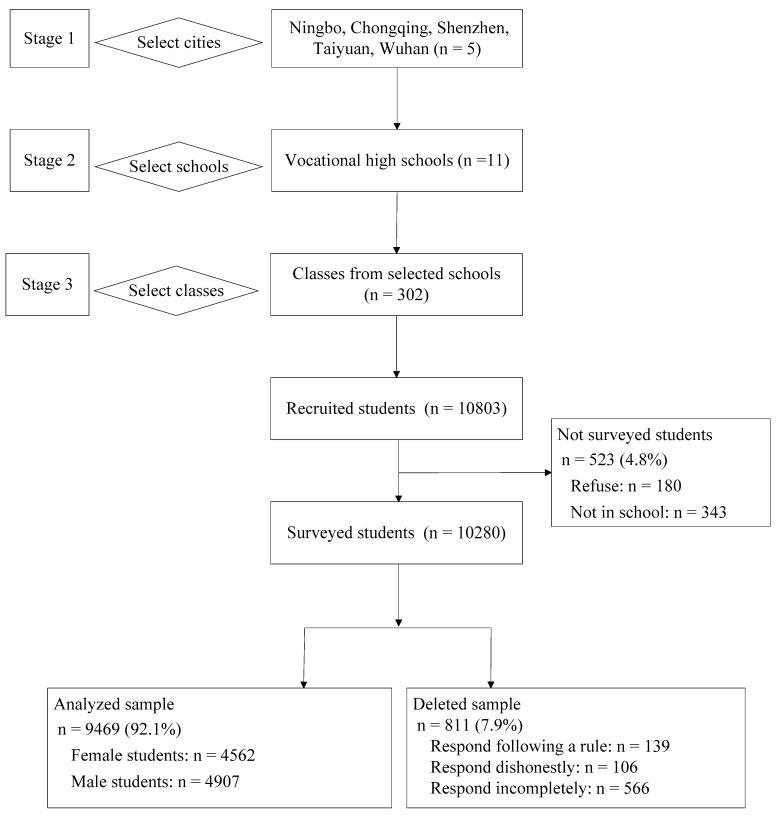
Flow chart for sampling process in the present study.

Data were collected from September 2013 to December 2014 among Chinese secondary vocational students. A multistage cluster sampling strategy (see Figure 1) was utilized to select a nationally representative sample. In stage 1, five metropolises (i.e., Ningbo, Chongqing, Shenzhen, Taiyuan and Wuhan) were purposively selected from the eastern, western, southern, northern and central areas of China, respectively. In stage 2, eleven secondary vocational schools were purposively selected from five cities, including three schools from Wuhan and two schools in each of the other four cities. In stage 3, students from all or randomly selected classes in each school were recruited to participate in the survey. Only students in the 10th and 11th grade were recruited because students in the 12th grade were not in school due to internship. Overall, a total of 10803 students were recruited from all 302 selected classes. There were 523 students (4.8%) excluded, because 180 students (1.7%) refused, and 343 students (3.2%) were not in school. This resulted in 10,280 students participating in the survey, yielding a response rate of 95.2%. A total of 9469 respondents (92.1%) provided valid data in this sample, which consisted of 4562 female students and 4907 male students, with an average age of 17 years.

### 2.2. Measures

In the present study, a battery of questions including self-reported club drug use, sociodemographic variables, individual factors, as well as peer and family related factors, were completed by students. Club drug use was measured by the following questions with five options (1 = never, 2 = tried them, but do not use them now, 3 = a few times monthly, 4 = a few times weekly, 5 = daily): How often (if ever) do you use the drugs listed below? These drugs included Ketamine, MA, MDMA, ‘Magu’ pills and GHB [37]. The lifetime club drug use in the present study was defined as “ever used any of these five drugs in their lifetime.”

Sociodemographic variables included gender (female/male), age (dichotomized into <18 or ≥18 years), ethnicity (coded into the Han Chinese or Minorities), grade (including the 10th and 11th grade), residence (coded into rural or urban) and living with parents (coded into yes or no). Social economic status (SES) was evaluated only by the occupations of parents. There were two questions separately asking paternal and maternal occupation with twelve options, which were then categorized into five levels (1 to 5), with a higher value indicating higher SES [38]. We chose a higher value from the responses of two questions to represent SES, which was finally coded into low (level 1), medium (level 2–4) and high (level 5).

Part-time job experience was measured by “Have you ever done a part-time job more than one month?” with a response of yes/no. Expenses per month were categorized into <1000 or ≥1000 Yuan. Academic achievement was measured by “what was your average grades last semester?”, with responses coded into three levels (<60, 60–79, ≥80). Smoking behavior was measured by “How often (if ever) do you smoke a cigarette?”, with the same five options as club drug use. Lifetime smoking was coded into yes/no.

Social benefit expectancies assessed positive beliefs about club drug use with seven items [39,40], e.g., “Adolescents who use club drugs have more friends”. Responses were described as below: 1 = strongly disagree, 2 = disagree, 3 = neither disagree nor agree, 4 = agree, 5 = strongly agree. The answer was coded as a 0 score if students chose responses 1 or 2, while responses 3 to 5 were coded into a 1 score. Then, perceiving social benefit expectancies towards club drugs was dichotomized into yes (total score ≥ 1) or no (total score = 0).

Refusal skills were assessed by “How likely would you be able to use skills as following when someone offers you a club drug?”. Five kinds of skills were included, i.e., directly saying ‘no’, telling them you do not want to use it, changing the subject, suggesting other activities, as well as making up an excuse and leaving [41]. Each skill was responded to with a 5-point scale from 1 (definitely would) to 5 (definitely would not). The answer was coded into 1 score if students chose response “definitely would”, and other responses were coded into 0 score. Then, the variable refusal skills were coded into none (total score = 0), medium (total score = 1–4) and high (total score = 5).

Based on the previous study [42], peer drug use was measured by “How many of your friends use club drugs?”, with five options ranging from 1 (none) to 5 (almost all). Perceiving peer’s approval of drug use was measured by “How do you think the attitude of your friends if you try to use club drugs?” with five options ranging from 1 (strongly disapprove) to 5 (strongly approve). Family drug use was measured by “How many of your family members use club drugs?” with the same options as peer drug use. The responses of these three variables were finally coded into yes or no.

### 2.3. Ethics Statement and Data Collection

The study was reviewed and approved by the Medical Ethics Committee of Tongji Medical College, Huazhong University of Science and Technology and we obtained Institutional Review Board (IRB) approval for the conduct of this study. Furthermore, at each of the eleven participating schools, we obtained agreement from the principals before we conducted the survey, given that all of the participating students were in a young age group and that most of them were remote from their parents or guardians. Moreover, before paper-and-pencil questionnaires were distributed, all students in selected classes were told that they could quit the survey whenever they wanted. One well-trained investigator collected the data in each class within one class time (40 min). The confidentiality and anonymity were stressed before students began to answer the questionnaires. They were told that none of their parents, teachers, friends and classmates would know any related information. The class teachers were absent from the classroom during the survey. All materials were anonymous and in Chinese.

### 2.4. Statistical Analysis

We used a double-entry strategy to enter all data into Epidata Version 3.1 (The EpiData Association, Odense, Denmark). Questionnaires were all checked with two quality control strategies before data entry. One strategy was to assess false reports through a question in the end of questionnaires to ask whether students honestly answered the items. Data would be eliminated if participants did not respond honestly. The other one was to assess the attitude of students. If students answered the questions by following a rule, such as choosing the same options for most items, the data would be discarded. After data entry, questionnaires with incomplete responses were also excluded from the analyses. Overall, a total of 811 respondents (7.9%) were deleted according to these strategies.

Data were analyzed using SPSS version 20.0 (SPSS Inc., Chicago, IL, USA) and STATA 16. The characteristics of each variable and the prevalence of club drug use were explained. In order to understand any different relationship between associated factors and club drug use by gender, data were analyzed for female and male students separately. Univariate and multivariable Poisson regression models with robust variance were conducted to explore the factors associated with club drug use. All statistically significant variables in the univariate analyses were adjusted in the multivariable analyses. Unadjusted and adjusted prevalence ratios (PRs) and 95% confidence intervals (CIs) were obtained in the regression models [43,44]. All hypothesis tests were 2-tailed, and the significance level was set at α = 0.05.

## 3. Results

### 3.1. Characteristics of Total Respondents

As shown in Table 1, male and 10th grade students accounted for 51.8% and 57.9% in the present study. About 6.9% of the included students were 18 years old or above. The Han Chinese accounted for 97.4% of the total respondents. Most of the students were from urban areas (67.5%), lived with parents (76.9%) and spent less than 1000 Yuan per month (87.5%). Slightly more than half of students got an academic achievement between 60–79 scores (53.7%), had medium SES (55.1%) and high levels of refusal skills for club drugs (51.5%). Approximately one-third of students had ever smoked (34.7%) and had part-time job experience (35.8%). Nearly a quarter of students (22.3%) perceived social benefit expectancies towards club drugs. A minority students reported their friends (7.6%) and family members (3.6%) used club drugs. Moreover, 3.6% of students reported their friends would approve them if they used club drugs. Additionally, the overall lifetime prevalence of club drug use was 2.7% and male students had higher prevalence than female students (3.5% vs. 1.9%, *χ^2^* = 21.03, *p* < 0.001).

### 3.2. Univariate Poisson Regression Analyses

The univariate Poisson regression analyses results were shown in Table 1. Without adjusting for other variables, ethnicity, academic achievement, expense per month, lifetime smoking, perceiving social benefit expectancies, refusal skills, peer drug use, perceiving peer’s approval of drug use and family drug use were all associated with club drug use among female and male students. However, age, SES and the experience of part-time work were only associated with club drug use among male students.

### 3.3. Multivariate Poisson Regression Analyses

The multivariable Poisson regression analyses results were presented in Table 2. After controlling for statistically significant variables from univariate Poisson regression analyses, five common variables were all retained in final models for both female and male students. Students who had ever smoked and those perceiving social benefit expectancies were more likely to use club drugs. Moreover, peer drug use and perceiving peer’s approval of drug use could increase the risk of club drug use for students. Conversely, students who had medium or high levels of refusal skills were less likely to use club drugs. Furthermore, family using club drugs and having a part-time job experience were two additionally independent risk factors of club drug use among male students.

## 4. Discussion

The current study is a nationally representative cross-sectional survey to understand the prevalence of and factors associated with club drug use among Chinese secondary vocational students. The data were separately analyzed for female and male students. As shown in results, the overall lifetime prevalence of five club drugs (i.e., Ketamine, MA, MDMA, ‘Magu’ pills and GHB) was 2.7% among Chinese secondary vocational students and some independent associated factors (five for female students and seven for male students) were determined from 15 indicators.

Even though many other studies reported a wider overall prevalence of illicit drugs use (including traditionally used drugs like marijuana, heroin and cocaine), we found that the lifetime prevalence of secondary vocational student’s club drug use in our study was also higher than that from previous national [45,46] and regional [33,34,36] surveys in China. Two reasons might cause these differences. One is that the illicit drugs, especially club drugs, are more and more prevalent among Chinese younger people nowadays [15]. The other is that previous studies [33,34] involved more extensive student samples which also included elementary, junior or senior high school students. A series of Chinese studies [33,34,46] had shown that secondary vocational students had a higher prevalence of illicit drugs use than other student population in school.

Peer influence is usually a leading risk factor for adolescent substance use [36,47,48,49]. We found the similar result that peer drug use had the strongest relationship with club drug use among female (aPR = 3.00) and male (aPR = 4.98) students, respectively. Meanwhile, perceiving peer’s approval of drug use was consistently found as a risk factor for student’s club drug use (aPR = 2.01 and 1.94 for female and male students, respectively). Individuals who have friends using drugs, or approving them to use drugs, might get more chance to obtain drugs and learn the same behavior from these peers [31]. Furthermore, they share similar beliefs, attitudes, values, and rationales for drug use, which could also prompt them to use drugs [47].

Positive outcome expectancies have been theoretically and empirically corroborated its significant influence on promoting drug use [29,36,50]. In the current study, our results were in accordance with these findings. Perceiving social benefit expectancies consistently increased the risk of club drug use for female (aPR = 1.96) and male (aPR = 2.40) students. This finding might be explained by a general expectancy-based model of substance use development [50]. The model elucidated that positive expectancies could motivate the initial substance use. Then, the experience of substance use may help reinforce expectancies in memory and further promote drug-taking behaviors. Meanwhile, we also found that refusal skills were taken as a protective factor for club drug use among female and male students. This was in agreement with previous studies [29,36]. High levels of refusal skills could help students to resist the peer and social pressure to use drugs [48]. Therefore, refusal skills have been typically considered as necessary social and cognitive skills in drug use prevention programs [48,51,52]. Furthermore, smoking was usually considered as the gateway behavior for illicit drug use [53]. Consistent with prior studies [11,12,31,54], lifetime smoking was an independent risk factor among secondary vocational student’s club drug use in our study (aPR = 2.27 and 2.51 for female and male students, respectively).

Additionally, some gender differences were found in the current study. First, our study consistently showed that male students had higher lifetime prevalence of club drug use than female students [11,13,31,33]. In China, licit drugs (e.g., smoking and drinking) use was socially accepted and usually regarded as a symbol of independence and social status among males [55,56], and males might exhibit lower levels of self-control than females [57], all of which might increase the risk of illicit drugs use among males. Second, the type of associated factors had some distinction between different genders. As shown in the results, except for five common associated factors, family drug use (aPR = 1.39) and having a part-time job experience (aPR = 1.53) had additional independent risk effects on club drug use among male students. The observation of a family member using drugs not only offers an example for students to model [9,50], but also promotes them forming positive expectancies towards club drug use, which thereby increases the use of club drugs [51]. The location of a part-time job was usually outside schools, in which people were more likely to communicate an acceptable attitude for drug use. This would lead to students having a higher risk of exposure to other drug users and increase the opportunities for students to use licit or illicit drugs [58].

This study has some limitations. First, the cross-sectional design made it difficult for causal inference. Longitudinal studies should be conducted to verify the current findings. Second, it might be unavoidable that students misreported the status of drug use. However, the confidentiality and anonymity were stressed to promote the cooperation of students and other quality control strategies like checking questionnaires carefully had been taken in the study. Third, the prevalence of each club drug was not reported, and some variables such as sensation seeking [31] and circadian rhythm [28], which might have the far-reaching influence on club drug use, were not involved in the present study. Consequently, further studies targeting specific drugs and involving a wider range of associated factors should be considered. Finally, according to the previous theory framework [59], some mediation or moderation effects might exist among determinants in our study, but these are beyond the scope of this article. Further work should be considered to explore these effects in the future.

## 5. Conclusions

In summary, the prevalence of and factors associated with club drug use among Chinese secondary vocational students were assessed by a nationally representative sample in our study. Some independent risk and protective factors at individual, peer and family levels were separately determined for female and male students. These findings provide important implications on club drug use prevention for Chinese secondary vocational students. Peer education might be an excellent approach for the salient influence of peer factors. Moreover, it is very important to educate family members. Correcting inaccurate beliefs and promoting refusal skills might be beneficial to reduce club drug use. Smoking, as a gateway behavior to club drug use, should be addressed ahead of, or synchronous with, club drug use prevention. Moreover, the experience of a part-time job should also be considered. Furthermore, gender difference is a significant influential factor worthy of attention as well. Many prevention programs involving these associated factors have demonstrated short and long-term effects on adolescent drug use in other countries (especially in western countries) [48,60]. Nonetheless, only a limited number of prevention programs have been established in China [51,52]. Therefore, there is an urgent need for more programs to prevent the use of illicit drugs to be developed for Chinese students and our present study provides important information for this work.

## Figures and Tables

**Table 1:** Demographic characteristics of the total sample, unadjusted prevalence ratios (PR) and 95% confidence intervals (CIs) of lifetime club drug use among female and male students.

Variables		Total (*n* = 9469)	Female Students (*n* = 4562)			Male Students (*n* = 4907)		
		n (%)	Club Drug Use (%)	Unadjusted PR (95% CI)	*p*	Club Drug Use (%)	Unadjusted PR (95% CI)	*p*
Club drugs use		258 (2.7)	88 (1.9)			170 (3.5)		
Ethnicity	Han	9223 (97.4)	81 (1.8)	1 (Reference)		160 (3.3)	1 (Reference)	
	Minorities	246 (2.6)	7 (5.3)	2.92 (1.38−6.20)	0.005	10 (8.7)	2.60 (1.37−4.93)	0.003
Age	<18 years	8819 (93.1)	82 (1.9)	1 (Reference)		143 (3.2)	1 (Reference)	
	≥18 years	650 (6.9)	6 (2.1)	1.11 (0.49−2.53)	0.795	27 (7.3)	2.32 (1.54−3.50)	<0.001
Grade	10th	5478 (57.9)	55 (2.2)	1 (Reference)		104 (3.4)	1 (Reference)	
	11th	3991 (42.1)	33 (1.6)	0.70 (0.45−1.07)	0.098	66 (3.5)	1.02 (0.75−1.39)	0.895
Residence	Rural	3075 (32.5)	30 (2.1)	1 (Reference)		58 (3.6)	1 (Reference)	
	Urban	6394 (67.5)	58 (1.9)	0.90 (0.58−1.39)	0.632	112 (3.4)	0.96 (0.70−1.32)	0.790
Living with parents	No	2184 (23.1)	28 (2.7)	1 (Reference)		45 (4.0)	1 (Reference)	
	Yes	7285 (76.9)	60 (1.7)	0.65 (0.41−1.01)	0.053	125 (3.3)	0.83 (0.59−1.17)	0.281
Academic achievement	<60	1079 (11.4)	15 (5.5)	1 (Reference)		39 (4.8)	1 (Reference)	
	60~79	5082 (53.7)	43 (1.8)	0.32 (0.18−0.57)	<0.001	82 (3.1)	0.64 (0.44−0.93)	0.020
	≥80	3308 (34.9)	30 (1.6)	0.29 (0.16−0.54)	<0.001	49 (3.4)	0.70 (0.46−1.07)	0.100
Part-time job	No	6081 (64.2)	54 (1.8)	1 (Reference)		77 (2.5)	1 (Reference)	
	Yes	3388 (35.8)	34 (2.2)	1.20 (0.78−1.83)	0.403	93 (5.1)	2.05 (1.52−2.78)	<0.001
Social economic status (SES)	Low	1381 (14.6)	15 (2.2)	1 (Reference)		36 (5.1)	1 (Reference)	
	Medium	5222 (55.1)	43 (1.7)	0.75 (0.42−1.34)	0.330	76 (2.9)	0.56 (0.38−0.84)	0.005
	High	2866 (30.3)	30 (2.3)	1.01 (0.55−1.87)	0.964	58 (3.8)	0.74 (0.49−1.12)	0.158
Expense per month (Yuan)	<1000	8287 (87.5)	63 (1.6)	1 (Reference)		128 (3.0)	1 (Reference)	
	≥1000	1182 (12.5)	25 (4.8)	3.04 (1.93−4.80)	<0.001	42 (6.4)	2.13 (1.50−3.01)	<0.001
Lifetime smoking	No	6180 (65.3)	37 (1.0)	1 (Reference)		36 (1.4)	1 (Reference)	
	Yes	3289 (34.7)	51 (5.2)	4.98 (3.28−7.56)	<0.001	134 (5.8)	4.22 (2.92−6.10)	<0.001
Perceiving social benefit expectancies	No	7354 (77.7)	38 (1.0)	1 (Reference)		55 (1.5)	1 (Reference)	
	Yes	2115 (22.3)	50 (5.4)	5.17 (3.41−7.84)	<0.001	115 (9.7)	6.53 (4.74−9.01)	<0.001
Refusal skills	None	496 (5.2)	26 (16.0)	1 (Reference)		60 (18.0)	1 (Reference)	
	Medium	4095 (43.2)	35 (1.8)	0.12 (0.07−0.19)	<0.001	67 (3.0)	0.17 (0.12−0.24)	<0.001
	High	4878 (51.5)	27 (1.1)	0.07 (0.04−0.11)	<0.001	43 (1.8)	0.10 (0.07−0.15)	<0.001
Peer drug use	No	8754 (92.4)	52 (1.2)	1 (Reference)		71 (1.6)	1 (Reference)	
	Yes	715 (7.6)	36 (12.7)	10.43 (6.94−15.68)	<0.001	99 (23.0)	14.48 (10.68−29.64)	<0.001
Perceiving peer’s approval of drug use	No	9126 (96.4)	70 (1.6)	1 (Reference)		117 (2.5)	1 (Reference)	
	Yes	343 (3.6)	18 (20.2)	12.92 (8.05−20.75)	<0.001	53 (20.9)	8.30 (6.00−11.48)	<0.001
Family drug use	No	9132 (96.4)	65 (1.5)	1 (Reference)		121 (2.6)	1 (Reference)	
	Yes	337 (3.6)	23 (16.0)	10.86 (6.95−16.95)	<0.001	49 (25.4)	9.89 (7.10−13.78)	<0.001

**Table 2:** Adjusted prevalence ratios (aPR) and 95% confidence intervals (CIs) of lifetime club drug use among female and male students.

Variables		Female Students (*n* = 4562)		Male Students (*n* = 4907)	
		Adjusted PR (95% CI)	*p*	Adjusted PR (95% CI)	*p*
Ethnicity	Han	1 (Reference)		1 (Reference)	
	Minorities	1.41 (0.65−3.07)	0.390	1.86 (0.95−3.64)	0.070
Age	<18 years			1 (Reference)	
	≥18 years			1.29 (0.85−1.94)	0.230
Academic achievement	<60	1 (Reference)		1 (Reference)	
	60~79	0.70 (0.37−1.31)	0.264	1.14 (0.83−1.57)	0.421
	≥80	0.59 (0.32−1.08)	0.088	1.07 (0.75−1.54)	0.697
Part-time job	No			1 (Reference)	
	Yes			1.53 (1.16−2.01)	0.003
Social economic status (SES)	Low			1 (Reference)	
	Medium			0.72 (0.51−1.02)	0.062
	High			0.84 (0.59−1.21)	0.354
Expense per month (Yuan)	<1000	1 (Reference)		1 (Reference)	
	≥1000	1.18 (0.75−1.85)	0.476	1.17 (0.85−1.59)	0.337
Lifetime smoking	No	1 (Reference)		1 (Reference)	
	Yes	2.27 (1.42−3.64)	0.001	2.51 (1.73−3.63)	<0.001
Perceiving social benefit expectancies	No	1 (Reference)		1 (Reference)	
	Yes	1.96 (1.22−3.16)	0.005	2.40 (1.70−3.40)	<0.001
Refusal skills	None	1 (Reference)		1 (Reference)	
	Medium	0.32 (0.17−0.58)	<0.001	0.45 (0.32−0.64)	<0.001
	High	0.24 (0.12−0.45)	<0.001	0.40 (0.27−0.61)	<0.001
Peer drug use	No	1 (Reference)		1 (Reference)	
	Yes	3.00 (1.75−5.15)	<0.001	4.98 (3.39−7.30)	<0.001
Perceiving peer’s approval of drug use	No	1 (Reference)		1 (Reference)	
	Yes	2.01 (1.10−3.70)	0.024	1.94 (1.46−2.59)	<0.001
Family drug use	No	1 (Reference)		1 (Reference)	
	Yes	1.48 (0.79−2.77)	0.218	1.39 (1.01−1.92)	0.042

## Data Availability

The data presented in this study are available on request from the corresponding author. The data are not publicly available due to confidentiality of participants.
